# Correlation between three assay systems for anti-Müllerian hormone (AMH) determination

**DOI:** 10.1007/s10815-012-9880-1

**Published:** 2012-11-02

**Authors:** Hang Wun Raymond Li, Ernest Hung Yu Ng, Benancy Po Chau Wong, Richard A. Anderson, Pak Chung Ho, William Shu Biu Yeung

**Affiliations:** 1 Department of Obstetrics & Gynaecology, Queen Mary Hospital, The University of Hong Kong, 102 Pokfulam Road, Hong Kong, People’s Republic of China; 2 MRC Centre for Reproductive Health, The University of Edinburgh, Royal Infirmary of Edinburgh, 51 Little France Crescent, Edinburgh, EH16 4SA Scotland, UK

**Keywords:** Anti-Mullerian hormone, ELISA kit

## Abstract

**Purpose:**

Analysis of anti-Müllerian hormone (AMH) is becoming of recognized importance in reproductive medicine, but assays are not standardized. We have evaluated the correlation between the new Gen II ELISA kit (Beckman-Coutler) and the older ELISA kits by Immunotech (IOT) and Diagnostic Systems Laboratories (DSL).

**Methods:**

A total of 56 archived serum samples from patients with subfertility or reproductive endocrine disorders were retrieved and assayed in duplicate using the three AMH ELISA kits . The samples covered a wide range of AMH concentrations (1.9 to 142.5 pmol/L).

**Results:**

We observed good correlations between the new (AMH Gen II) and old AMH assay kits by IOT and DSL (R^2^ = 0.971 and 0.930 respectively). The regression equations were AMH (Gen II) = 1.353 × AMH (IOT) + 0.051 and AMH (Gen II) = 1.223 × AMH (DSL) – 1.270 respectively.

**Conclusions:**

AMH concentrations using the Gen II kit are slightly higher than those from the IOT and DSL kits. Standardization of assay results worldwide is urgently required but this analysis facilitates the interpretation of values obtained historically and in future studies using any of the 3 assays available. Meanwhile, adapting clinical cut-offs from previously published work by direct conversion is not recommended.

## Introduction

Anti-Müllerian hormone (AMH), also known as Müllerian-inhibiting substance, is a dimeric glycoprotein that belongs to the transforming growth factor-beta family. It is involved in the regression of the Müllerian ducts during male fetal development [12]. In the female, AMH is solely produced by the granulosa cells of preantral and small antral follicles, and regulates ovarian activity and follicular steroidogenesis. Because of this exclusive source of production in the adult female, AMH is a potentially useful marker of ovarian function, and there have been increasing reports on its clinical utility [1].

Because of its stable production throughout the menstrual cycle with clinically insignificant intra- and inter-cycle variation [9, 20], and that its serum concentration is not influenced by the use of exogenous hormones [16], AMH may serve as a clinically useful diagnostic tool in differentiating the different causes of secondary oligo-amenorrhoea with very good diagnostic accuracy as well as its utility in the research context. Serum AMH concentration is significantly increased by 2 to 3 times in women with WHO group 2 anovulation including polycystic ovary syndrome (PCOS), and is significantly reduced to very low levels in premature ovarian failure (POF), while remaining unchanged in hypogonadotrophic hypogonadism and hyperprolactinaemia compared to ovulatory women [10, 17].

In in-vitro fertilization (IVF) treatment cycles, basal serum AMH level is significantly correlated with the number of follicles obtained after stimulation and the number of retrieved oocytes, and is useful in prediction of suboptimal and excessive ovarian responses upon ovarian stimulation [1, 3, 4, 18]. Although most reports indicate a poor predictive value of AMH on pregnancy rate in the fresh cycle of IVF treatment, as reviewed in a recent meta-analysis [3], it has been suggested that it does give some prediction of pregnancy [11], particularly when taking both the fresh and frozen embryo transfers into account [7, 14]. In controlled ovarian stimulation and intrauterine insemination, a recent study also showed that higher serum AMH level was associated with significantly higher cumulative live birth rate [15].

Worldwide, two commercially available ELISA kits for determination of AMH have been available, manufactured by Diagnostic Systems Laboratories, Inc. (DSL) and Immunotech (IOT), both under the umbrella of Beckman Coulter, Inc. Recently, a new ELISA kit, called “AMH Gen II ELISA”, has been marketed by Beckman Coulter to replace the two older kits. According to information from the manufacturer, the AMH Gen II ELISA kit uses the same antibody as in the DSL kit but the standards of the IOT assay kit [8].

Guidance is therefore required to interpret the growing literature derived from using the different assay systems available. The different assays have been discussed [19] but no studies have directly compared the 3 available assay systems on the same sample set. We carried out this analysis to evaluate the correlation between the new and old AMH ELISA kits by running parallel assays on the same batch of serum samples using the three kits mentioned, so as to find out the correlation between the results of the old and new kits, and to validate the new kit for future clinical and research utility.

## Methods

We analysed a total of 56 serum samples taken from patients suffering from polycystic ovary syndrome or subfertility (with causes including male factor, tubo-peritoneal factor, endometriosis and unexplained subfertility) who attended the Edinburgh Fertility and Reproductive Endocrine Centre, Edinburgh, United Kingdom with informed, written consent and local ethical committee approval. Parallel assays were repeated using the three AMH ELISA kits as follows:

1. Active MIS/AMH ELISA (catalogue number DSL-10-14400) (DSL, Webster, TX, USA), which has a sensitivity of 0.04 pmol/l, and reported intra- and inter-assay coefficients of variation of less than 4.6 % and 8.0 % respectively according to the product insert;
2. EIA AMH/MIS (catalogue number A16507) (IOT, Marseille, France), which has a sensitivity of 0.7 pmol/l, and reported intra- and inter-assay coefficients of variation of less than 12.3 % and 14.2 % respectively according to the product insert;
3. AMH Gen II (catalogue number A79765) (Beckman Coulter, Chaska, MN, USA), which has a sensitivity of 0.57 pmol/l, and reported intra- and inter-assay coefficients of variation of less than 5.4 and 5.6 % respectively according to the product insert.

All samples were assayed in duplicate on all the three kits.

Correlation of AMH concentrations determined by the different kits were analysed by the Passing and Bablok regression model using the MedCalc version 11.6 software.

## Results

Comparative analysis was performed on a total of 56 serum samples. The median age of the subjects was 31.5 (range 18.6–40.4) years. The AMH concentration ranged from 1.9 to 142.5 pmol/l based on the Gen II kit.

**Fig. 1:**
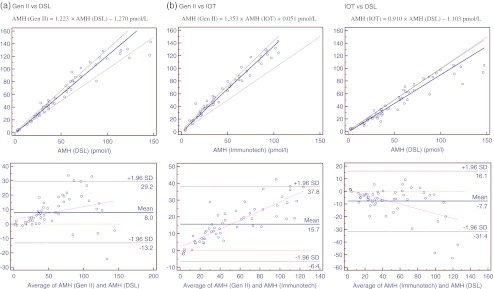
Correlation between three commercial ELISA kits for determination of anti-Mullerian hormone (AMH). The upper panels represent the Passing and Bablok regression whereas the lower panels represent the Bland-Altman plot

The respective scatter plot diagrams with the curve fit, as well as the Bland-Altman plots are shown in Fig. 1. There were good correlations of the Gen II kit with both DSL (R^2^ = 0.930, *p* < 0.0001) and IOT kits (R^2^ = 0.971, *p* < 0.0001). On the Passing and Bablok regression model, the regression equation correlating the Gen II and DSL kits was: AMH (Gen II) = 1.223 × AMH (DSL) –1.270 pmol/L, while that correlating the Gen II and IOT kits was: AMH (Gen II) = 1.353 × AMH (IOT) + 0.051 pmol/L. The regression equation correlating the IOT and DSL kits was: AMH (IOT) = 0.910 × AMH (DSL) –1.103 pmol/L (R^2^ = 0.906, *p* < 0.0001). All three regression curves showed no significant deviation from linearity (*p* > 0.05). The Bland-Altman plots confirmed that the values obtained with the Gen II kit were systematically higher than those from the IOT and DSL kits.

It was observed that the difference between values obtained from two kits tended to be smaller and the dispersion of the difference tended to be greater in samples with AMH values greater than 100 pmol/L. If only samples with AMH values of less than 100 pmol/L in the X-axis were analysed, the following regression equations are obtained:

$$ \begin{array}{*{20}c} {\mathrm{AMH}\left( {\mathrm{Gen}\ \mathrm{II}} \right)=1.376\times \mathrm{AMH}\left( {\mathrm{IOT}} \right)--0.679\ \mathrm{pmol}/\mathrm{L}} \\ {\mathrm{AMH}\left( {\mathrm{Gen}\ \mathrm{II}} \right)=1.330\times \mathrm{AMH}\left( {\mathrm{DSL}} \right)--4.174\ \mathrm{pmol}/\mathrm{L}} \\ {\mathrm{AMH}\left( {\mathrm{IOT}} \right)=0.973\times \mathrm{AMH}\left( {\mathrm{DSL}} \right)--2.963\ \mathrm{pmol}/\mathrm{L}} \\ \end{array} $$

## Discussion

Our results demonstrate excellent correlation between the new Gen II kit and both the DSL and IOT kits. This is reassuring and allows general interpretation of data obtained using all 3 systems. According to our regression equations, AMH values obtained with the new assay kit will be some 35 % and 22 % higher than with the IOT and DSL kits respectively. As revealed in the Bland-Altman plots, the variations in differences between the Gen II and the older kits appeared to be greater at higher AMH values. However, cut-offs for clinical utility are mostly at the lower range of values and hence variations at the higher end are probably of less clinical importance. When only samples with AMH values less than 100 pmol/L were taken, the Gen II kit gave values about 38 % and 33 % higher than with the IOT and DSL kits respectively.

Two previous studies have compared the Gen II kit to the DSL [22] and IOT kits [8]. The former gave a regression equation: Gen II = 1.40 × DSL –0.62 pmol/L (R^2^ = 0.9216, *n* = 271, with AMH concentration ranging from 0 to about 100 pmol/l), while the latter gave a regression equation: Gen II = 1.0018 × IOT ng/ml (R^2^ = 0.98, *n* = 120, with AMH concentration ranging from 0–20 ng/ml i.e. 0–143 pmol/l). Thus both our finding and that by Wallace et al. [22] revealed higher values given by the Gen II assay than the DSL assay, and both reported comparable conversion factors at AMH values of less than 100 pmol/L. The Gen II kit is stated to be calibrated to the IOT kit and yet we observed 35 % higher values with the Gen II kit, in contrast to that reported by Kumar et al. [8]. The reason for this discrepancy is not certain.

There are also several reports on comparison of AMH values between the IOT and DSL kits [2, 5, 6, 13, 21]. Strikingly, these show widely different regression equations, with both lower and comparable AMH values with the DSL kit compared with the IOT kit being reported. In contrast, our findings revealed a slightly higher instead of lower value with the DSL kit compared with the IOT kit.

A point to note is that this analysis was based on the unit pmol/l. Users who adopt the unit ng/ml for AMH can refer to the conversion formula: 1 ng/ml = 7.18 pmol/l.

Based on these discrepant findings among studies, it is obvious that an international standard should be urgently developed before widespread clinical applications of AMH assays are further developed. We do not recommend adapting clinical cut-offs from previously published work by direct conversion. Instead, it may be more appropriate to redefine such cut-off values for clinical application by further studies using a unified international standard. As the only commercial ELISA kit for AMH assay at the moment replacing the two older ones, the AMH Gen II ELISA kit could potentially serve this role. Certainly an automated system, which could further improve the efficiency and precision of the assay, is awaited. When these are available, further validation studies will be required to determine universal standardised cut-off values for various clinical applications.

In conclusion, we have shown a good correlation between the new and old AMH assay kits. The introduction of the new kit appears to be a useful substitute for the two old assay kits, taking into account the altered calibration. However universal standardization of assay results worldwide and the determination of clinically meaningful cut-off values is essential for the robust and widespread use of AMH in clinical and research practice.
